# HyperPhS: a pharmacophore-guided multimodal representation framework for metabolic stability prediction through contrastive hypergraph learning

**DOI:** 10.1093/bioinformatics/btaf524

**Published:** 2025-09-22

**Authors:** Xiaoyi Liu, Na Zhang, Chenglong Kang, Chengwei Ai, Hongpeng Yang, Jijun Tang, Fei Guo

**Affiliations:** School of Chinese Materia Medica, Beijing University of Chinese Medicine, Beijing 100029, China; Computer Science and Engineering, Central South University, Changsha 410083, China; Computer Science and Engineering, Central South University, Changsha 410083, China; Computer Science and Engineering, Central South University, Changsha 410083, China; Computer Science and Engineering, University of South Carolina, Columbia, SC 29208, United States; Computer Science and Engineering, Shenzhen University of Advanced Technology, Nanshan 518055, China; Computer Science and Engineering, Central South University, Changsha 410083, China

## Abstract

**Motivation:**

Metabolic stability is crucial in the early stage of drug discovery and development. Drug candidate screening and optimization can be streamlined through the accurate prediction of stability. Functional groups within drug molecules are known as pharmacophores, which bind directly to receptors or biological macromolecules to produce biological effects, thereby affecting metabolic stability. Therefore, determining metabolic stability via the pharmacophore groups remains a significant challenge.

**Results:**

To address these issues, we propose a Pharmacophore-guided Hypergraph representation framework for predicting metabolic Stability (HyperPhS). In this study, we introduce a hypergraph-based method to extract features from metabolic pharmacophores with multi-view representation and contrastive learning. In particular, we introduce a pharmacophore-based contrastive learning encoder that captures the consistency between functional and nonfunctional structures. Our method applies ChatGPT simultaneously to metabolites and heterogeneous encoders and integrates multimodal representations by using attention-driven fusion modules coupled with fully connected neural networks. On the HLM dataset, HyperPhS achieves outstanding performance with 87.6% in AUC and 62.6% in MCC, alongside an external test AUC of 88.3%. In addition, pharmacophore groups studied by HyperPhS are validated for their interpretability through case studies. Overall, HyperPhS is an effective and interpretable tool for determining metabolic stability, identifying critical functional groups, and optimizing compounds.

**Availability and implementation:**

The code and data are available at https://github.com/xiaoyiliu-usc/HyperPhS.

## 1 Introduction

Metabolic stability, which refers to how extensively and quickly a drug is metabolized in the body, is crucial in the early stages of drug discovery and development (Rao Gajula *et al.* 2021). It significantly impacts key pharmacokinetic parameters, such as oral bioavailability, volume of distribution, clearance, half-life, and toxicity. These factors are essential for determining the appropriate dosage and frequency of drug administration (Liu *et al.* 2023). Thus, improving the metabolic stability of hit and lead compounds early in the drug discovery process is essential for effective drug candidate screening and lead optimization (Mao *et al.* 2023).

Metabolic stability is traditionally assessed through *in vitro* studies, where early-stage compounds are incubated with Liver Microsomes (LMs) to evaluate their metabolism (Gajula *et al.* 2021). LMs are abundant in cytochrome P450 (CYP450) enzymes crucial for drug metabolism and are widely employed in preliminary pharmacokinetic (PK) evaluations (Słoczyńska *et al.* 2019). Mouse, rat, and human LMs (MLMs, RLMs, HLMs) play a key role in these studies. However, these methods are often time-consuming, labor-intensive, and costly.

Recent advancements in machine learning have introduced various models to predict metabolic stability. Perryman *et al.* (2016) developed a Bayesian model and Ryu *et al.* (2022) applied a random forest model with HLM data. However, these models often underperform due to the overlooking of the molecular structure. Therefore, Renn *et al.* (2021) used Graph Convolution Networks (GCNs) to convert molecular SMILES sequences into graph structures, and Du *et al.* (2023) employed a graph contrastive learning strategy with Gated Recurrent Units (GRU) to integrate SMILES and topological features, enhancing prediction accuracy. However, the vastness of chemical space makes these methods difficult to scale and requires complex computation.

A pharmacophore is a functional group within a metabolic molecule that binds to receptors or biological macromolecules to produce biological effects (Gao *et al.* 2010). Effectively incorporating pharmacophore-guided key groups, rather than the entire molecular graph of metabolites, minimizes computational demands (Zhu *et al.* 2023). However, the effective integration of pharmacophore information remains a significant challenge. For example, current pharmacophore-based models suffer from limited representational capacity, often reducing pharmacophores to low-order or pairwise features (Jiang *et al.* 2023). These approaches fail to capture the high-dimensional, group-based structural dependencies that influence metabolic activity (Li *et al.* 2022a).

**Figure 1.**
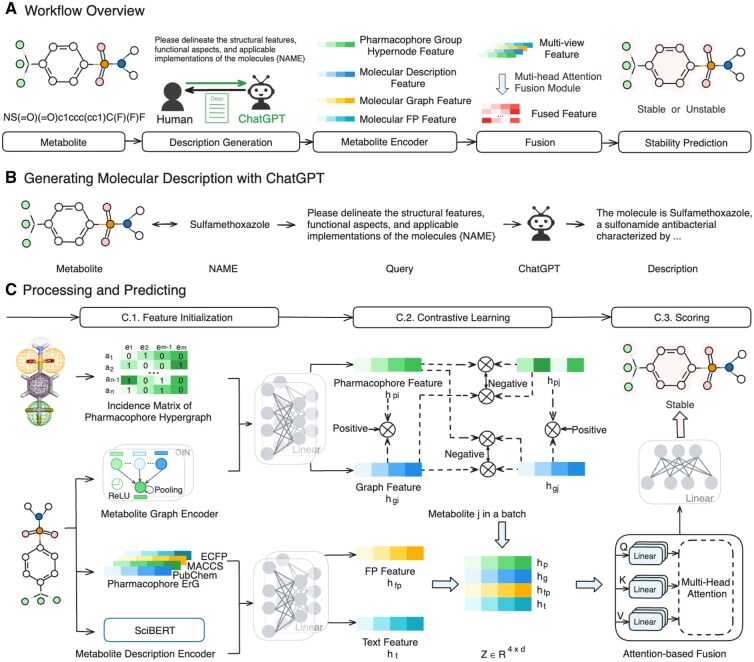
An overview of HyperPhS. (A) Metabolic stability prediction workflow. (B) Generating metabolite descriptions with ChatGPT. (C) Pharmacophore-guided feature initialization. (C.1) Multi-view feature encoding using hypergraphs, GINs, fingerprints, and SciBERT. (C.2) Contrastive learning between graph and hypergraph representations. (C.3) An attention-based module for feature fusion and a multilayer perceptron (MLP) for stability prediction.

To address these challenges, we propose a pharmacophore guided multi-view representation for metabolic stability prediction through contrastively hypergraph learning, named HyperPhS (as shown in Fig. 1). HyperPhS represents pharmacophores as hyperedges connecting multiple atoms, enabling high-order structural learning and seamless integration with graph and text modalities in a unified contrastive framework. Specifically, HyperPhS leverages a text-assisted module that uses language models, including ChatGPT, to generate descriptions of metabolites from their SMILES representation and integrates these descriptions with molecular multimodal representations to improve stability prediction. To further enhance the learning of multiple distinct feature data sets, the HyperPhS model incorporates contrastive learning alongside specially designed metabolite encoders to effectively capture various structural aspects. Moreover, HyperPhS employs attention-based mechanisms for combining the information from different views of compounds in the fusion module. As a result, this model can identify and combine the most relevant information from each view. Finally, HyperPhS is then developed as a metabolic stability predictor.

Overall, the main contributions can be summarized as follows:

1. We propose HyperPhS, a novel hypergraph contrastive learning model that accurately predicts metabolic stability while providing interpretability by identifying key pharmacophores.
2. We design a multi-view learning framework that introduces pharmacophore data alongside molecular structures and fingerprints to learn comprehensive features for metabolic stability prediction.
3. HyperPhS outperforms state-of-the-art (SOTA) methods on two benchmark datasets, while case studies of stable and unstable molecules further validate its utility by emphasizing the critical relationship between pharmacophores and metabolic stability.

## 2 Materials and methods

### 2.1 Problem description

We formulate the metabolic stability prediction problem as a binary classification task. Given a set of *N* compounds, denoted as $D=\{D_{i}|i\in 1,\ldots ,N\}$, along with a corresponding set of metabolic stability labels $Y=\{y_{i}|y_{i}\in 0,1,i\in 1,\ldots ,N\}$ for each compound $D_{i}$, where $y_{i}=1$ indicates that the *i*th compound is metabolically stable and $y_{i}=0$ indicates it is unstable, our objective is to train a classifier $f_{\theta }$ to assign probability scores $\hat{y}$ to the compounds, predicting their stability. Here, θ represents the classifier parameters.

### 2.2 Overview of HyperPhS

In this study, we propose a novel pharmacophore-guided hypergraph learning framework called HyperPhS (as shown in Fig. 1). HyperPhS effectively captures pharmacophore information through hypergraph learning and focuses on structural and nonstructural subgraphs using contrastive learning to reduce computation. Specifically, HyperPhS’s text-assisted module utilizes large language models, such as ChatGPT, to generate descriptions of metabolites based on their SMILES representation, which are integrated with molecular graphs for stability prediction. Additionally, HyperPhS utilizes metabolite encoders to learn distinct metabolite representations in parallel, extracting features from four key sources: metabolite descriptions, pharmacophore-guided hypergraphs, molecular graphs, and fingerprints. We also introduce an attention-based fusion module to integrate these multi-view representations, allowing the model to identify and combine the most relevant information from each view. Finally, the stability predictor assesses the stability of a given molecular structure based on the learned representations. We will provide a detailed introduction to each module.

### 2.3 Compound encoder

#### 2.3.1 Molecule description and representation module

In this study, ChatGPT is employed to generate metabolite descriptions. Chemical structures are uniquely represented by International Union of Pure and Applied Chemistry (IUPAC) names, which follow a set of rules that map structures to more natural, language-like phrases compared to SMILES (Weininger 1988). Specifically, for all metabolites, SMILES strings were first converted into their corresponding IUPAC names using the PubChemPy library. These IUPAC names were subsequently used as input to ChatGPT-4 for generating textual descriptions, employing the following prompt:

*Please delineate the structural features, functional aspects, and applicable implementations of the molecules* {*NAME*}*. And commence with the introduction: “The molecule is …” and reply to me in a sentence.*

where {*NAME*} corresponds to the IUPAC name of the metabolite.

SciBERT (Beltagy *et al.* 2019) is used as the $\mathrm{Encode}r_{t}$ for the textual modality (Fig. 1B).


(1) $$h_{t}=\mathrm{Encode}r_{t}(T),$$

where $T=[t^{1},\ldots ,t^{l}]$ denotes the text input, *l* is the text length.

#### 2.3.2 Pharmacophore-guided hypergraph representation module

A pharmacophore hypothesis comprises multiple chemical features and their spatial relationships. We use *Basefeatures.fdef* from RDKit (Landrum 2006). Based on the definitions of molecular substructures and their corresponding pharmacophore features in Zhu *et al.* (2023), we extracted all pharmacophore features for each molecule. Common pharmacophore features include aromatic rings, cations (positive charge center), hydrogen bond acceptors, and hydrogen bond donors. Hydrophobic groups are also common and include hydrophobes and lumped hydrophobes. Rare features are unknown. Supplementary Information, available as supplementary data at *Bioinformatics* online provides a detailed description of the pharmacophore hypergraph and its preparation.

To describe the complex relationships between pharmacophores in a molecule, we utilized a hypergraph convolution network and attention module to treat each pharmacophore as a fully integrated subgraph. We also learned deep embeddings on high-order graph-structured data, as introduced in Feng *et al.* (2019). Since a hyperedge can connect more than two vertices, each one represents a pharmacophore and connects the corresponding atoms. For the pharmacophores of a given molecule, let hypergraph $H_{p}=\{V,E\}$, with vertices $V=\{v_{1},v_{2},\ldots ,v_{n}\}$ and hyperedges $E=\{e_{1},e_{2},\ldots ,e_{m}\}$, $e_{i}\subseteq V$. The hypergraph $H_{p}$ can be represented by its incidence matrix $H\in R^{n\times m}$. Each element of the matrix is a Boolean value that indicates whether an atom is associated with a particular pharmacophore. This can be obtained from the stoichiometric matrix by converting its non-zero values into binary ones through binarization. The number of atoms is denoted by *n* while the number of pharmacophores is represented by *m*.

For a hypergraph incidence matrix H, each hyperedge ϵ is assigned a positive weight $W_{\epsilon \epsilon }$, with all weights stored in the diagonal matrix $W\in R^{m\times m}$. The vertex degree matrix $D_{ii}=\sum_{\epsilon =1}^{m}W_{\epsilon \epsilon }H_{i\epsilon }$ represents the number of hyperedges containing each vertex. Similarly, the hyperedge degree matrix $B_{\epsilon \epsilon }=\sum_{i=1}^{n}H_{i\epsilon }$.

The hypergraph convolution operation for updating vertex embeddings, with row normalization applied to achieve directional and asymmetric propagation is shown in Equation (2):


(2) $$X^{\left(l+1\right)}=\delta (D^{-1}HWB^{-1}H^{T}X^{\left(l\right)}P),$$

where $X^{\left(l\right)}\in R^{n\times F^{\left(l\right)}}$ and $X^{\left(l+1\right)}\in R^{n\times F^{\left(l+1\right)}}$ represent the input to layers (l) and (l+1), respectively. $P\in R^{F^{\left(l\right)}\times F^{\left(l+1\right)}}$. Matrices D and B are the vertex and hyperedge degree matrices, respectively.

To get the molecule hypergraph representation $h_{p}$, we apply global max-pooling and mean-pooling to the atom representations $X^{\left(l\right)}$ and concatenate the resulting vectors


(3) $$h_{p}=\text{CONCAT}\left(\max\{X^{\left(l\right)}\},\text{mean}\{X^{\left(l\right)}\}\right),$$

#### 2.3.3 Graph representation module

The molecular graph, representing the relationships between atoms and bonds, captures both local and global structure (Tsubaki *et al.* 2019, Cai *et al.* 2022). We introduce the Graph Isomorphism Network (GIN) to learn molecular representations from these graphs. For a molecule *i*, its graph G has nodes (atoms) and edges (bonds), with adjacency matrix A. GIN iteratively aggregates neighboring atom features, with each atom’s representation $h_{v}^{\left(k\right)}$ in layer *k* defined as:


(4) $$h_{v}^{\left(k\right)}=\text{MLP}\left(\left(1+\alpha^{\left(k\right)}\right)\cdot h_{v}^{\left(k-1\right)}+\sum_{u\in N(v)}h_{u}^{\left(k-1\right)}\right),$$

where $\alpha^{\left(k\right)}$ is a learnable parameter and N(v) represents neighbors of node *v*. After *K* iterations, $h_{v}$ encodes *K*-hop neighborhood information.

To obtain the molecule-level representation $h_{g}$, we apply global max and mean-pooling to atom representations $h_{v}$ and concatenate the results:


(5) $$h_{g}=\text{CONCAT}\left(\max\{h_{v}(u)\},\text{mean}\{h_{v}(u)\}\right),$$

#### 2.3.4 Fingerprint representation module

Compound fingerprints are binary vectors indicating the presence or absence of specific substructures and are categorized into substructure key-based, topological or path-based, and circular fingerprints (Jia *et al.* 2020). In this study, SMILES notations were converted into four fingerprint types—Pharmacophore ErG, PubChem, MACCS, and Extended Connectivity FingerPrint (ECFP) using the RDKit toolkit (Landrum 2006). Firstly, pharmacophore-type node descriptions are used to generate a 441-bit Pharmacophore ErG fingerprint via the ErG method (Stiefl *et al.* 2006). Secondly, we apply a 166-bit MACCS fingerprint (Durant *et al.* 2002), which encodes atomic and bond characteristics using SMARTS patterns. Thirdly, an 881-bit PubChem fingerprint is employed for broad chemical structure coverage. Finally, circular fingerprints are generated using the 1024-bit ECFP with the Morgan algorithm, with a maximum neighborhood diameter of 2. After concatenating the fingerprint vectors, an MLP with non-linear activation functions, such as ReLU, is applied to obtain a fingerprint representation, denoted as $h_{fp}\in R^{d}$:


(6) $$h_{fp}=\text{MLP}(fp_{\mathrm{pub}}||fp_{\mathrm{maccs}}||fp_{\mathrm{erg}}||fp_{\mathrm{ecfp}}),$$

### 2.4 Contrastive learning

Inspired by research showing that graph representation learning is improved by maintaining consistency between local and global structures (You *et al.* 2020). Therefore, we introduce graph contrastive learning to enhance the representation learning of metabolites.

Suppose we randomly sample a mini-batch of *Q* molecular graphs and derive an equal number of the corresponding hypergraphs. We adopt pharmacophore-guided hypergraphs via contrastive learning to enhance learning. In particular, for a given molecule *i*, its graph representation and its hypergraph representation, while its representation and the representations of the remaining 2(Q−1) graphs/hypergraphs form negative pairs. The primary idea of graph contrastive learning is to maximize the agreement between the representations of positive pairs while minimizing the agreement between the representations of negative pairs (Chen *et al.* 2020).

For a given molecule *i*, its graph and hypergraph representations ($h_{g}$ and $h_{p}$) are passed through an MLP projection head producing $z_{i}$ and ${\hat{z}}_{i}$ which are used for calculating the contrastive loss:


(7) $$\ell_{z_{i},{\hat{z}}_{i}}^{\text{contr}}=-\text{log}\;\frac{\;\text{exp}\;\left(\text{sim}(z_{i},{\hat{z}}_{i})/\tau \right)}{\sum_{k=1,k\neq i}^{2Q}\;\text{exp}\;\left(\text{sim}(z_{i},z_{k})/\tau \right)},$$

where $\text{sim}(z_{i},{\hat{z}}_{i})=\frac{z_{i}^{T}{\hat{z}}_{i}}{\left||z_{i}||||{\hat{z}}_{i}|\right|}$ denotes the similarity between the representations $z_{i}$ and ${\hat{z}}_{i}$, i.e. cosine similarity. τ is the temperature parameter (set to 0.2 by default).

### 2.5 Attention-based fusion module

Our proposed fusion module utilizes the attention mechanism to effectively merge the multimodal features of the metabolites. The attention mechanism is capable of assigning different weights to the molecular features from different views, effectively highlighting the most informative and relevant features for a metabolic stability prediction task (Xiong *et al.* 2019, Wu *et al.* 2023).

Firstly, the features from different views are concatenated as a matrix $Z=[h_{t};h_{p};h_{g};h_{fp}]\in R^{4\times d}$. Then, three-parameter matrices $W^{Q}\in R^{d\times d_{k}}$, $W^{K}\in R^{d\times d_{k}}$, and $W^{V}\in R^{d\times d_{v}}$ are utilized to compute the matrices for Query $Q=ZW^{Q}$, Key $K=ZW^{K}$, and Value $V=ZW^{V}$ matrices.

The attention mechanism is utilized to effectively weigh the different multi-view representations of a molecule in our proposed method. By computing the dot products of the Q and K matrices, dividing by $\sqrt{d_{k}}$, and applying the softmax function to obtain the attention weight matrix from the values as shown in Equation (8)


(8) $$\mathrm{Attention}(Q,K,V)=\mathrm{softmax}(\frac{QK^{T}}{\sqrt{d_{k}}})V,$$

To enable our model to focus on information from different views simultaneously, HyperPhS incorporates multi-head attention. The resulting output matrix is shown in Equation (9):


(9) $$\mathrm{hea}d_{i}=\mathrm{Attention}(ZW_{i}^{Q},ZW_{i}^{K},ZW_{i}^{V}),$$

For each head, we get the representation $\mathrm{hea}d_{i}\in R^{4\times d_{v}}$, and then feed the representation into a 4×4 convolution layer (denoted as Conv()) to incorporate these features selectively. Finally, the features from *M* heads are concatenated as the output features for a compound:


(10) $$h_{c}=\text{CONCAT}\left(\mathrm{Conv}(\mathrm{hea}d_{i}),\ldots ,\mathrm{Conv}(\mathrm{hea}d_{M})\right),$$

### 2.6 Metabolic stability predictor

Furthermore, the outputs of the multi-head attention layers are fed into an MLP layer to get the predicted score $\hat{y}$ for each metabolite:


(11) $$\hat{y}=\mathrm{sigmoid}(\text{MLP}(h_{c})),$$

Finally, Binary Cross-Entropy (BCE) loss is employed to compute the loss between the predicted vector $\hat{y}$ and the ground truth vector y. The overall loss function is defined as:


(12a) $$L^{C}=-\sum_{i=1}^{Q}[y_{i}\;\text{log}({\hat{y}}_{i})+(1-y_{i})\log(1-{\hat{y}}_{i})],$$

(12b) $$L^{CL}=\frac{1}{2Q}\sum_{i=1}^{Q}\left(\ell_{z_{i},{\hat{z}}_{i}}^{\text{contr}}+\ell_{\hat{z_{i}},z_{i}}^{\text{contr}}\right),$$

(12c) $$\mathrm{Loss}=L^{C}+L^{CL}.$$

## 3 Results

### 3.1 Benchmark dataset and evaluation metrics

Table 1 summarizes the Human Liver Microsomes (HLM), Rat Liver Microsomes (RLM), and external datasets used in our experiments, sourced from Li *et al.* (2022b). Model performance was assessed via 10-fold cross-validation on the HLM and RLM datasets, as well as an independent test consisting of training on the HLM dataset and testing on the external dataset, with results averaged over 10 runs. Key evaluation metrics included accuracy, *F*1 score, area under the curve (AUC), and Matthews correlation coefficient (MCC).

**Table 1.** Statistics of metabolite dataset.

Dataset	HLM	HLM external	RLM	RLM external
Positive (stable)	3784	82	1542	736
Negative (unstable)	2094	29	1566	1746
Total	5878	111	3108	2482

### 3.2 Implementation details

Our model incorporates four complementary views of compounds, including text-based molecular descriptions, graph-based structural representations, pharmacophore-guided hypergraphs capturing higher-order interactions, and fingerprint-based substructure encodings.

For the text-based view, we used the GPT-4 model via the OpenAI API, and the SciBERT (Beltagy *et al.* 2019) is downloaded from Hugging Face. For the graph-based view, the initial node feature dimension is set to 84, and two GIN layers are used. The pharmacophore-guided hypergraph has two hypergraph layers followed by four attention heads. For the fingerprint view, each molecule is represented by a 2513-dimensional binary feature vector, with embedding obtained through a fully connected layer with 512 neurons. Four MLPs map the different representations to the same dimension of 512 to fuse the multi-view embeddings. The fusion module uses four attention heads to weigh the four views of a compound, such as metabolite descriptions, pharmacophore-guided hypergraphs, molecular graphs, and molecular fingerprints with attention dimensions $d_{k}$ and $d_{v}$ set to 2048. The τ is the temperature parameter set to 0.2 by default for the contrastive learning process. Moreover, HyperPhS is optimized using Adam (Kingma and Ba 2014) with a learning rate of 0.0005, 200 epochs, a batch size of 256, a dropout rate of 0.5, a data augmentation ratio of 0.4, and ReLU as the activation function. Furthermore, HyperPhS is implemented in Python 3.8 and PyTorch 2.0, utilizing functionalities from PyG 2.3.1, TensorFlow 2.13.0, Transformers 4.44.0, NetworkX 2.8.4, scikit-learn 1.2.2, numpy 1.23.5, pandas 2.0.2, and RDKit 2023.03.1.

### 3.3 Comparison with state-of-the-art methods

In this section, we evaluate the performance of the proposed HyperPhS model by comparing it with nine SOTA methods, including three traditional machine learning-based and four deep learning-based models. We used the parameter settings recommended in the original references. Specifically, FP-GBDT and FP-XGBoost (Li *et al.* 2022b) used ECFP fingerprints, with FP-GBDT having a depth of 4 and 1200 trees, and FP-XGBoost having a depth of 3 and 900 trees. PredMS (Ryu *et al.* 2022) employed molecular descriptors with Random Forest-based selection using 500 trees. GCN (Renn *et al.* 2021) used a two-layer architecture, while GAT (Veličković *et al.* 2017) had two layers with two attention heads. D-MPNN (Li *et al.* 2022b) incorporated atomic and bond features with six message-passing iterations, followed by two feed-forward layers. AttentiveFP (Xiong *et al.* 2019) used a three-layer message-passing network. CMMS-GCL (Du *et al.* 2023) utilized graph contrastive learning to integrate molecular graph and sequence representations for metabolic stability prediction. The MS-BACL model (Wang *et al.* 2024) employed bond graph augmentation and contrastive learning for stability prediction to complete understanding of molecular structures.

#### 3.3.1 Performance evaluation on benchmark dataset

Tables 2 and 3 show the results of 10-fold cross-validation on the HLM and RLM datasets. For the HLM dataset, as shown in Table 2, our model (HyperPhS) achieves the best performance in all metrics, with an AUC of 87.6%±0.017%, an accuracy of 83.0%±0.018%, an *F*1 score of 87.0%±0.021%, and a MCC of 62.6%±0.039%. The results also show that HyperPhS improved the prediction performance on MCC by more than 11% compared to the GCN and GAT models. For example, HyperPhS improved the AUC, accuracy, *F*1 score, and MCC by 1.1%, 1.9%, 1.4%, and 6%, respectively, compared to the CMMS-GCL model. Additionally, HyperPhS significantly increased the accuracy of prediction performance by 1.0% and MCC by 2.5% compared to MS-BACL.

**Table 2.** Performance of HyperPhS compared to baseline methods for HLM metabolic stability prediction.^a^

Method	AUC	Accuracy	*F*1 score	MCC
FP-GBDT (Li *et al.* 2022b)	0.815	0.773	0.830	0.503
XGBoost (Li *et al.* 2022b)	0.844	0.793	0.846	0.548
D-MPNN (Li *et al.* 2022b)	0.842	0.792	0.841	0.541
GAT (Veličković *et al.* 2017)	0.858	0.782	0.842	0.533
PredMS (Ryu *et al.* 2022)	0.854	0.785	0.843	0.552
MGCN (Renn *et al.* 2021)	0.852	0.784	0.825	0.544
AttentiveFP (Xiong *et al.* 2019)	0.853	0.793	0.836	0.564
CMMS-GCL (Du *et al.* 2023)	0.865	0.811	0.856	0.566
MS-BACL (Wang *et al.* 2024)	0.873	0.820	0.863	0.601
**HyperPhS (ours)**	**0.876**	**0.830**	**0.870**	**0.626**

a The best results are highlighted in bold.

**Table 3.** Performance of HyperPhS compared to baseline methods for RLM metabolic stability prediction.^a^

Method	AUC	Accuracy	*F*1 score	MCC
FP-GBDT (Li *et al.* 2022b)	0.820	0.740	0.750	0.470
XGBoost (Li *et al.* 2022b)	0.810	0.710	0.720	0.420
D-MPNN (Li *et al.* 2022b)	0.840	0.750	0.760	0.500
GAT (Veličković *et al.* 2017)	0.789	0.788	0.789	0.580
PredMS (Ryu *et al.* 2022)	0.800	0.730	0.740	0.460
MGCN (Renn *et al.* 2021)	0.706	0.706	0.702	0.413
AttentiveFP (Xiong *et al.* 2019)	0.862	0.780	0.795	0.590
CMMS-GCL (Du *et al.* 2023)	0.866	0.783	0.778	0.570
MS-BACL (Wang *et al.* 2024)	0.866	0.783	0.778	0.570
**HyperPhS (ours)**	**0.888**	**0.806**	**0.807**	**0.612**

a The best results are highlighted in bold.

For the RLM dataset, as shown in Table 3, our model (HyperPhS) also achieves the best performance in all evaluation metrics, with an AUC of 88.8%, an accuracy of 80.6%, an *F*1 score of 80.7%, and an MCC of 61.2%. Compared to the CMMS-GCL model, HyperPhS shows improvements of 2.2% in AUC, 2.3% in accuracy, 2.9% in *F*1 score, and 4.2% in MCC. Additionally, compared to the MS-BACL model, HyperPhS achieves higher AUC, accuracy, *F*1 score, and MCC by 2.2%, 2.3%, 2.9%, and 4.2%, respectively. These results further highlight the ability of HyperPhS to provide more accurate and robust predictions of molecular metabolic stability compared to other models across different datasets.

Three main factors contribute to the superior performance of HyperPhS over baseline models. First, by incorporating features based on pharmacophores, HyperPhS captures critical chemical properties for metabolic stability, leading to more informative representations. Second, hypergraph contrastive learning allows the model to preserve both local and global molecular structures, enhancing representation learning. Third, the multi-view approach helps HyperPhS understand complex relationships within molecular structures, resulting in more accurate and robust predictions. These enhancements will be validated in the subsequent ablation study, demonstrating significant improvements in all evaluation metrics compared to other models.

#### 3.3.2 Independent evaluation on external dataset

To validate the effectiveness and generalizability of our model, we evaluated HyperPhS and other baselines on an external dataset, training on the HLM or RLM dataset and testing on the external dataset.

The results in Tables 4 and 5 demonstrate that HyperPhS achieved the best performance compared with nine methods across all metrics, based on the average results over 10 iterations. In the HLM-External dataset evaluation, HyperPhS achieved an AUC of 88.3%, an accuracy of 84.7%, an *F*1 score of 89.7%, and an MCC of 59.9%. Compared to CMMS-GCL (Du *et al.* 2023), HyperPhS provided a notable improvement in accuracy by 1.1% and MCC by 3.0%, showcasing its superior feature extraction capabilities. Additionally, HyperPhS demonstrated a higher MCC than MS-BACL (Wang *et al.* 2024), emphasizing its robustness in capturing intricate relationships within molecular structures.

**Table 4.** Performance of different models on the HLM-External dataset.^a^

Method	AUC	Accuracy	*F*1 score	MCC
FP-GBDT (Li *et al.* 2022b)	0.644	0.740	0.825	0.155
XGBoost (Li *et al.* 2022b)	0.678	0.732	0.830	0.150
D-MPNN (Li *et al.* 2022b)	0.766	0.760	0.852	0.218
GAT (Veličković *et al.* 2017)	0.814	0.755	0.825	0.414
PredMS (Ryu *et al.* 2022)	0.766	0.780	0.856	0.231
MGCN (Renn *et al.* 2021)	0.830	0.774	0.845	0.447
AttentiveFP (Xiong *et al.* 2019)	0.816	0.744	0.814	0.415
CMMS-GCL (Du *et al.* 2023)	0.885	0.836	0.889	0.569
MS-BACL (Wang *et al.* 2024)	0.897	0.842	0.895	0.588
**HyperPhS (ours)**	**0.883**	**0.847**	**0.897**	**0.599**

a The best results are highlighted in bold.

**Table 5.** Performance of different models on the RLM-External dataset.^a^

Method	AUC	Accuracy	*F*1 score	MCC
FP-GBDT (Li *et al.* 2022b)	0.630	0.610	0.690	0.190
XGBoost (Li *et al.* 2022b)	0.650	0.620	0.700	0.210
D-MPNN (Li *et al.* 2022b)	0.710	0.680	0.780	0.300
GAT (Veličković *et al.* 2017)	0.740	0.760	0.790	0.490
PredMS (Ryu *et al.* 2022)	0.600	0.560	0.640	0.110
MGCN (Renn *et al.* 2021)	0.680	0.690	0.740	0.360
AttentiveFP (Xiong *et al.* 2019)	0.850	0.780	0.790	0.590
CMMS-GCL (Du *et al.* 2023)	0.866	0.777	0.770	0.570
MS-BACL (Wang *et al.* 2024)	–	–	–	–
**HyperPhS (ours)**	**0.879**	**0.798**	**0.802**	**0.597**

a The best results are highlighted in bold.

Similarly, in the RLM-External dataset evaluation, HyperPhS outperformed the baseline methods, achieving an AUC of 87.9%, an accuracy of 79.8%, an *F*1 score of 80.2%, and an MCC of 59.7%. These improvements were particularly significant compared to models like GAT (Veličković *et al.* 2017) and CMMS-GCL (Du *et al.* 2023), indicating that HyperPhS’s incorporation of pharmacophore-based features and hypergraph contrastive learning effectively captured local and global structural information. The multi-view approach enhanced its ability to model complex molecular interactions, leading to more accurate predictions. Notably, the MS-BACL model (Wang *et al.* 2024) could not be successfully applied when trained on the RLM dataset and tested on the external dataset, as certain molecules could not be converted into bond graphs, thereby preventing the extraction of the corresponding features.

### 3.4 Ablation studies

To assess the contribution of various components in HyperPhS, we conducted ablation studies by removing key elements, with results summarized in Table 6. The graph-only variant (G-Model) achieved an AUC of 85.9%, accuracy of 79.6%, *F*1 score of 84.0%, and MCC of 0.561. Incorporating the hypergraph (GH-Model) improved performance slightly. The HyperPhS w/o Text variant, which excludes text features, performed better but the complete HyperPhS model, with text and fusion, outperformed all variants, achieving an AUC of 87.6%, accuracy of 83.0%, and MCC of 0.626. These results demonstrate that each component enhances predictive performance, including hypergraph contrastive learning, multi-view encoding, and attention-based fusion (see Supplementary Information Section 1, available as supplementary data at *Bioinformatics* online).

**Table 6.** Ablation studies of HyperPhS on HLM dataset.

Method	AUC	Accuracy	*F*1 score	MCC
G-model	0.859	0.796	0.840	0.561
GH-model	0.863	0.799	0.844	0.561
HyperPhS w/o text	0.873	0.809	0.853	0.585
HyperPhS w/o fusion	0.873	0.816	0.860	0.596
HyperPhS^a^	**0.876**	**0.830**	**0.870**	**0.626**

a The best results are highlighted in bold.

### 3.5 Performance assessment of novel structural molecules

To evaluate the ability of the model to predict the stability of molecules with novel structures, the Tanimoto similarity between molecules in the HLM dataset was calculated using their ECFP fingerprints, following the approach outlined in previous studies (Du *et al.* 2023, Wang *et al.* 2024). The molecules were clustered using the *K*-means algorithm into five groups based on structural similarity, and the clustering results were visualized through principal component analysis (PCA), see Fig. 1, available as supplementary data at *Bioinformatics* online.

**Figure 2.**
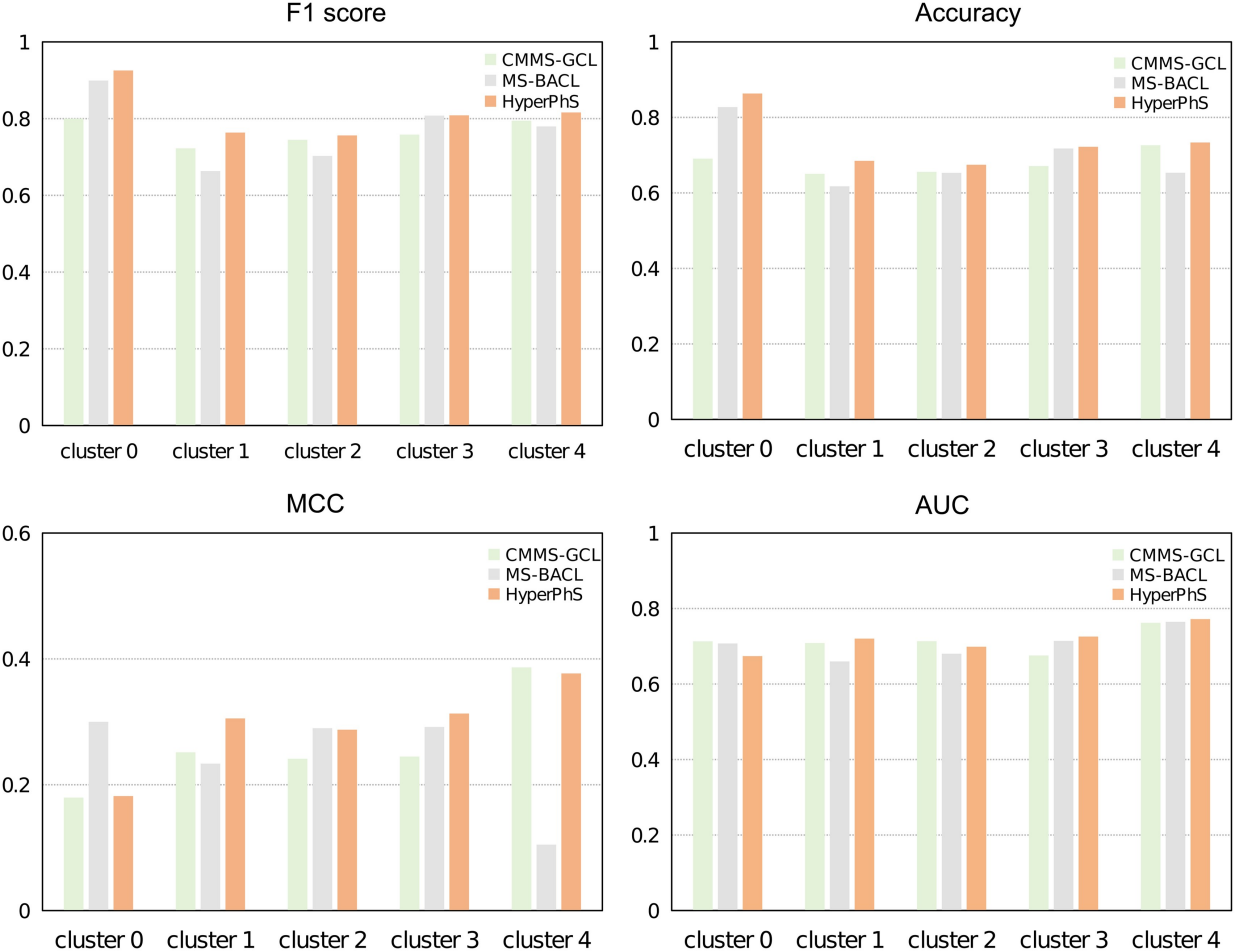
Performance of HyperPhS with suboptimal MS-BACL and CMMS-GCL models in identifying novel and diverse chemical structures.

Therefore, we conducted leave-one-cluster-out cross-validation, in which one cluster was used as the test set and the remaining four as the training set. This procedure ensured that molecules in the test set possessed novel structures. As shown in Fig. 2, the results indicate that our proposed model HyperPhS consistently outperformed the other two leading models across all five clusters. Specifically, HyperPhS showed superior *F*1 scores across all clusters, reaching 92.55% in cluster 0 and 81.61% in cluster 4, outperforming the baseline methods by up to 12.55% in cluster 0 compared to CMMS-GCL. Additionally, HyperPhS demonstrated the highest accuracy values in all clusters, including a 2.91% improvement over CMMS-GCL in cluster 4. This indicates that the model effectively handles known and novel molecular structures, highlighting its potential for applications in drug design.

### 3.6 Case study on interpretability

Following previous studies (Du *et al.* 2023, Wang *et al.* 2024), we conducted an in-depth analysis. This analysis focused on the stability of pharmacophore groups within molecular structures and their resulting impact on overall metabolic stability.

**Figure 3.**
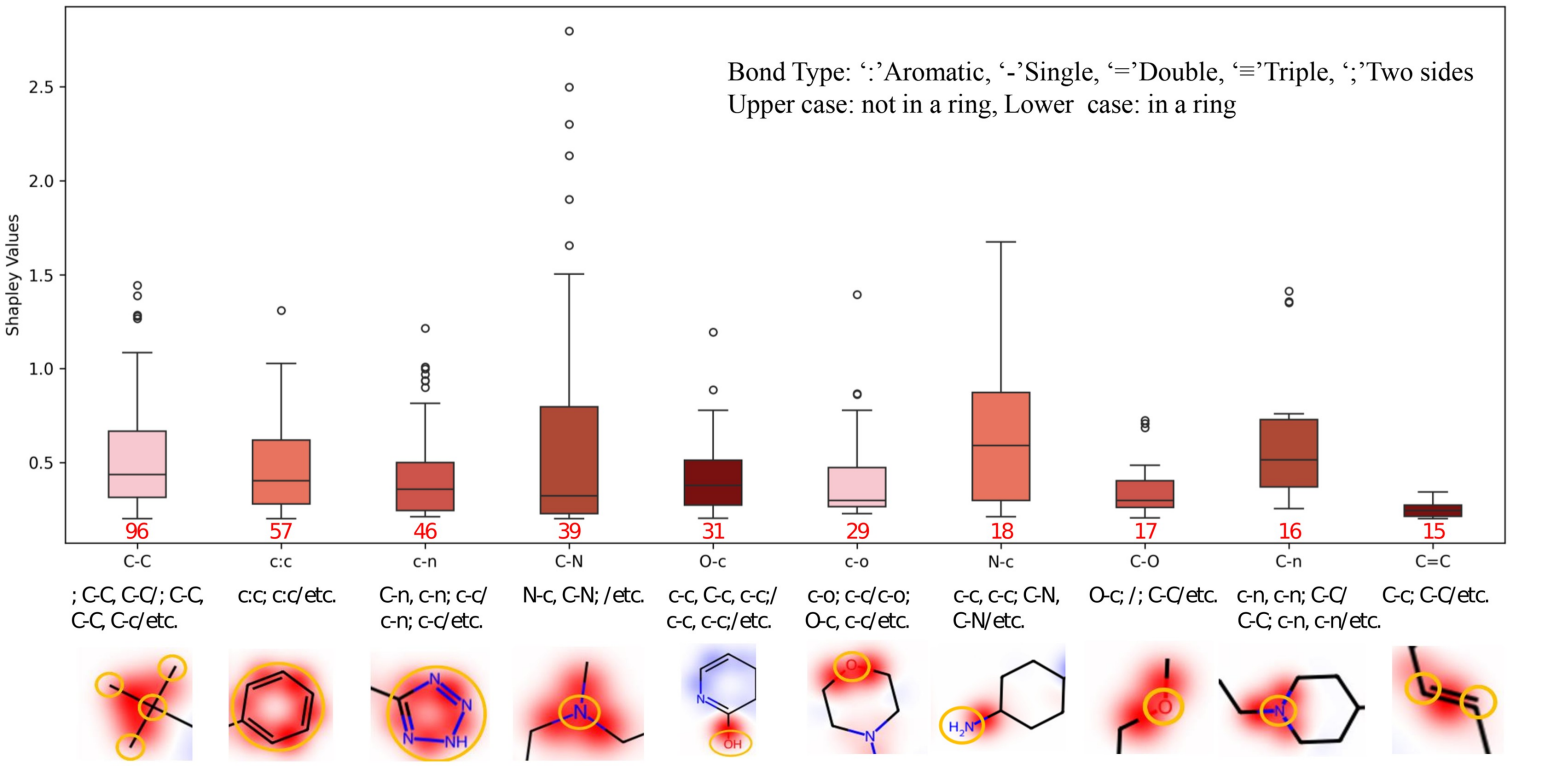
Case study on model interpretability, illustrating the top 10 functional groups influencing molecular stability, ranked by occurrence. Colors indicate positive (red) or negative (blue) impact, and relevant atoms are circled in orange.

To identify pharmacophore groups that significantly influence molecular stability, we set a Shapley value threshold of 0.2 as in the previous study (Du *et al.* 2023). Pharmacophore groups exceeding this threshold were considered critical for stability. We ranked these key pharmacophore groups and visualized the top 10 that occur the most frequently, as shown in Fig. 3. In the visualization, red indicates pharmacophore groups contributing positively to stability, while blue indicates groups contributing negatively, with the intensity of the color reflecting the degree of influence. The pharmacophore atoms are circled in orange (see Supplementary Information Section *Case study*, available as supplementary data at *Bioinformatics* online). We conducted experiments on 10 key functional groups (highlighted in orange on the molecular heatmap). Figure 2, available as supplementary data at *Bioinformatics* online, shows four stable molecules with highlighted stable pharmacophore regions, demonstrating the significant role of these groups in enhancing molecular stability.

In summary, our study successfully demonstrated the interpretability of the HyperPhS model, revealing that stable pharmacophore groups have a significant positive effect on molecular stability. Specifically, stable pharmacophores help maintain drug molecules’ structural integrity and functionality within a biological environment, thereby enhancing drug efficacy and safety. These findings provide new insights into the molecular mechanisms underlying stability and have important implications for drug design and chemical synthesis (Liu *et al.* 2024b).

## 4 Conclusion

In this work, we developed the HyperPhS model, utilizing multi-view learning with pharmacophore-guided hypergraph contrastive learning and an attention-based fusion module to predict molecular metabolic stability. Our results demonstrate that HyperPhS outperforms other models. Ablation studies confirm its strengths: it captures key chemical properties with pharmacophore-based features, improves understanding of molecular interactions via hypergraph learning, and preserves complex relationships through contrastive learning. Additionally, integrating multimodal learning with multiple metabolic encoders boosted performance across all metrics. In conclusion, HyperPhS enhances accuracy and interpretability in molecular stability prediction, offering valuable insights for drug discovery and metabolic network optimization (Mao *et al.* 2023, Liu *et al.* 2024a). Future work could further refine predictions by visualizing hypergraph relationships (Renn *et al.* 2021, Liu *et al.* 2023).

## Supplementary Material

btaf524_Supplementary_Data

## Data Availability

The data underlying this article are available in https://github.com/xiaoyiliu-usc/HyperPhS.
