# Supplementary material for: HyperPhS: a pharmacophore-guided multimodal representation framework for metabolic stability prediction through contrastive hypergraph learning
Source: Bioinformatics. 2025 Sep 22;41(10):btaf524. doi: 10.1093/bioinformatics/btaf524 (PMC12574321; doi:10.1093/bioinformatics/btaf524)
Supplement: btaf524_Supplementary_Data [file btaf524_supplementary_data.zip › Supplementary_Information.pdf]

Supplementary Materials

1 Ablation studies

To investigate the contribution of different components of HyperPhS to its predictive performance, we conducted ablation studies by systematically removing key elements of the model. The results of these ablation studies are summarized in Table Supplementary Table 1.

First, we evaluated the graph-only variant of HyperPhS, which exclusively leverages molecular graph features. This model achieved an AUC of 85.9%, an accuracy of 79.6%, an F1 score of 84.0%, and an MCC of 0.561. By comparing this variant to the full HyperPhS model, we observed that including the hypergraph and other feature types led to an improvement in all metrics. Specifically, HyperPhS outperformed the G-Model (Graph only from CMMS-GCL model) variant with an increase of 3.4% in AUC, 3.4% in accuracy, 3.0% in F1 score, and 6.5% in MCC. These results highlight the importance of incorporating additional views, such as hypergraph features, to enhance predictive performance.

Furthermore, we analyzed the impact of the pharmacophore-guided hypergraph representation module, denoted GH-Model (Graph + Hypergraph). This variant achieved slightly better performance compared to the G-Model, with an AUC of 86.3% and an accuracy of 79.9%. We also assessed the HyperPhS w/o Text variant, which uses graph, hypergraph, and fingerprint features but omits text-based features. This model showed improved results compared to the previous variants, with an AUC of 87.3%, an accuracy of 80.9%, and an MCC of 0.585. However, the complete HyperPhS model still outperformed this variant, showing the utility of text features in capturing important chemical properties that contribute to more accurate predictions. The HyperPhS w/o Fusion variant, which omits the attention-based fusion module, instead simply concatenates all feature types, achieved an AUC of 87.3%, an accuracy of 81.6%, an F1 score of 86.0%, and an MCC of 0.596. Although this variant performed reasonably well, it fell short of the complete HyperPhS model, which achieved an AUC of 87.6%, an accuracy of 83.0%, an F1 score of 87.0%, and an MCC of 0.626. This demonstrates the value of the attention-based fusion module, which effectively captures complex interactions among the different molecular features and leads to more robust prediction performance.

Overall, the ablation study results indicate that each component of HyperPhS contributes to enhancing the model's performance. Integrating hypergraph contrastive learning, multi-view feature encoding, and the attention-based fusion module led to substantial improvements. These findings underscore the importance of utilizing di-

verse molecular representations in conjunction with an advanced fusion strategy for accurately predicting molecular metabolic stability.

Supplementary Table 1: Ablation studies of HyperPhS on HLM dataset.

Method	AUC	Accuracy	F1 score	MCC
G-Model	0.859	0.796	0.840	0.561
GH-Model	0.863	0.799	0.844	0.561
HyperPhS w/o Text	0.873	0.809	0.853	0.585
HyperPhS w/o Fusion	0.873	0.816	0.860	0.596
HyperPhS	**0.876**	**0.830**	**0.870**	**0.626**

1.1 Principal Component Analysis Clusters

To evaluate the ability of the model to predict the stability of molecules with novel structures, the Tanimoto similarity between molecules in the HLM dataset was calculated using their ECFP fingerprint (Du *et al.*, 2023; Wang *et al.*, 2024). The molecules were clustered using the K-means algorithm into five groups based on structural similarity, and the clustering results were visualized through PCA.

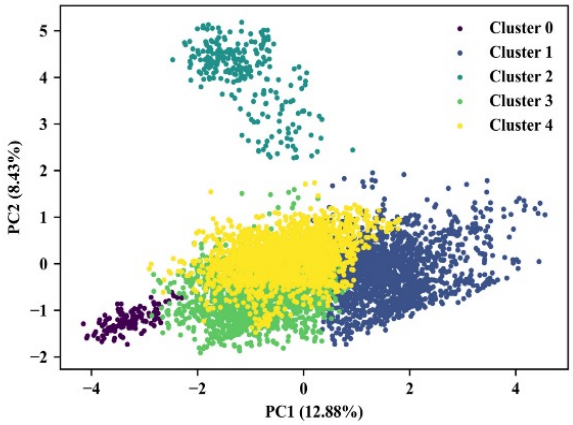

Supplementary Figure 1: PCA visualization of K-means clustering on the HLM dataset, where the values in parentheses correspond to the explained variance ratio.

References

Du, B.-X. *et al.* (2023). Cmms-gcl: cross-modality metabolic stability prediction with graph contrastive learning. *Bioinformatics*, **39**(8), btad503.

Wang, T. *et al.* (2024). Ms-bacl: enhancing metabolic stability prediction through bond graph augmentation and contrastive learning. *Briefings in Bioinformatics*, **25**(3), bbae127.
